# X-Ray Repair Cross-Complementing Group 1 (*XRCC1*) Genetic Polymorphisms and Risk of Childhood Acute Lymphoblastic Leukemia: A Meta-Analysis

**DOI:** 10.1371/journal.pone.0034897

**Published:** 2012-04-18

**Authors:** Libing Wang, Fan Yin, Xia Xu, Xiaoxia Hu, Dongbao Zhao

**Affiliations:** 1 Department of Hematology, Second Military Medical University, Changhai Hospital, Shanghai, China; 2 Department of Rheumatology and Immunology, Second Military Medical University, Changhai Hospital, Shanghai, China; IPO, Inst Port Oncology, Portugal

## Abstract

**Background:**

Recently, there have been a number of studies on the association between *XRCC1* polymorphisms and childhood acute lymphoblastic leukemia (ALL) risk. However, the results of previous reports are inconsistent. Thus, we performed a meta-analysis to clarify the effects of *XRCC1* variants on childhood ALL risk.

**Methods:**

A meta-analysis was performed to examine the association between *XRCC1* polymorphisms (Arg399Gln, Arg194Trp, and Arg280His) and childhood ALL risk. We critically reviewed 7 studies with a total of 880 cases and 1311 controls for Arg399Gln polymorphism, 3 studies with a total of 345 cases and 554 controls for Arg280His polymorphism, and 6 studies with a total of 783 cases and 1180 controls for Arg194Trp polymorphism, respectively. Odds ratio (OR) and its 95% confidence interval (CI) were used.

**Results:**

Significant association between *XRCC1* Arg399Gln polymorphism and childhood ALL risk was observed in total population analyses (OR_additive model_ = 1.501, 95% CI 1.112–2.026, P_OR_ = 0.008; OR_dominant model_ = 1.316, 95% CI = 1.104–1.569, P_OR_ = 0.002) and Asian subgroup analyses (OR_additive model_ = 2.338, 95%CI = 1.254–4.359, P_OR_ = 0.008; OR_dominant model_ = 2.108, 95%CI = 1.498–2.967, P_OR_ = 0.000). No association was detected in Caucasians, Metizo and mixed populations. Ethnicity was considered as a significant source of heterogeneity in the meta-regression model. For the other two *XRCC1* polymorphisms, no association with childhood ALL risk was found.

**Conclusions:**

The meta-analysis results suggested that *XRCC1* Arg399Gln polymorphism might be associated with elevated childhood ALL risk among Asian population.

## Introduction

While survival rates for childhood acute lymphoblastic leukemia (ALL) have been improved significantly over the past 50 years, ALL is still the most common pediatric cancer in developed countries with high incidence and mortality [1].

As a complex and multifactorial process, leukemogenesis is still not fully understood. It is widely known that dysregulated immune response to infection may be a cause of childhood ALL [2]. Although the role of environmental exposure is still currently undefined, it is likely that the environmental carcinogenesis exposure is influenced by co-inheritance of multiple low-risk variants, such as single nucleotide polymorphisms (SNPs) in susceptible genes [3]. These variants can be identified by comparing the frequency of polymorphic genotypes in cases and controls.

To date, the candidates for childhood ALL susceptibility genes have been categorized into those coding for carcinogen metabolism enzymes, folate metabolism enzymes, DNA repair proteins, and others [4]. The DNA repair system plays an important role in maintaining the genome integrity and stability through the reversal of DNA damage. If accumulated mutations are occurred in corresponding DNA repair genes, their reversal capacity could be damaged, substantially increasing the risk of cancer. SNPs in common DNA repair genes have been identified and demonstrated to be linked to several sporadic carcinogenesis [5], [6].

X-ray repair cross-complementing group 1 (*XRCC1*), located on chromosome 19q13.2–13.3, with 33 kilobases in length, is one of the most important proteins in base excision repair (BER) [7]. BER is also the predominant DNA damage repair pathway for the processing of small base lesions derived from oxidation and alkylation damage [8]. There have been more than 300 validated SNPs in the *XRCC1* gene reported in the dbSNP database (http://www.ncbi.nlm.nih.gov/SNP). Nevertheless, only three genetic changes have been extensively studied including Arg194Trp on exon 6 (rs1799782 in dbSNP, C/T), Arg280His on exon 9 (rs25489 in dbSNP, G/A), and Arg399Gln on exon 10 (rs25487 in dbSNP, G/A). There have been a number of studies on the association between *XRCC1* polymorphisms and childhood ALL risk [9]–[15]. However, these inconsistent results fail to clarify this complicated genetic relationship because of the small sample size and its low statistical power. To reliably demonstrate the effect of *XRCC1* variants (Arg399Gln, Arg280His, and Arg194Trp) on childhood ALL risk, we conduct a meta-analysis of all eligible studies to resolve this pivotal issue.

## Materials and Methods

### Study identification and selection

Computer searches of PubMed, EMBASE, Medline, Google Scholar and Cochrane Library were performed by two authors independently using the following key words (“childhood acute lymphoblastic leukemia” or ”childhood ALL”)and(“*XRCC1*” or “X-ray repair cross-complementation group 1”), covering all papers published before November 30, 2011. All eligible articles were retrieved and their references were searched simultaneously to find other relevant articles. Inclusion criteria was defined as follows: (1) case-control studies evaluating the association between *XRCC1* polymorphisms and childhood ALL risk; (2) studies based on unrelated individuals; (3) sufficient published data available to estimate an odds ratio (OR) with 95% confidence interval (CI). We excluded studies that were not full-length publications articles or letters in peer-reviewed English journals. When the same patient population was included in different articles, the one with the largest population of participants or the most recent one was selected.

### Data extraction

The following information was extracted from each study by two authors independently: first author, publication year, ethnicity, area, mean age of the study subjects, gender component, matching criteria, genotyping method, numbers of cases and controls, and genotype frequency of cases and controls. The two authors achieved a consensus at last.

### Statistical analysis

The strength of *XRCC1* polymorphisms and childhood ALL risk was assessed by odds ratios (ORs) with the corresponding 95% CI for each study. The OR and its 95% CI in each comparison were assessed in additive (aa versus AA; a was for the minor allele and A was for the major allele), dominant (aa+Aa versus AA), and recessive (aa versus Aa+AA) genetic models. Heterogeneity among studies was tested by chi-square-based Q test, and *I* ^2^ statistics was calculated to quantify the proportion of the total variation due to heterogeneity [16]. The pooled ORs were calculated by a fixed-effects model (the Mantel-Haenszel method) when the P value>0.05 for the Q test which indicated a lack of heterogeneity among the studies [17]. Otherwise, a random-effects model (DerSimonian-Laird method) was used [18]. The source of heterogeneity was explored in a meta-regression model. The significance of pooled ORs was determined by Z test (P<0.05 was considered statistically significant). The potential publication bias was examined visually in a funnel plot of log[OR] against its standard error (SE), and the degree of asymmetry was tested by Egger's test (P<0.05 was considered a significant publication bias) [19]. In the control populations, Hardy–Weinberg equilibrium (HWE) was tested. In addition, subgroup analysis for ethnicity (Asian, Caucasian, Mestizo, and Mixed population) was conducted, and influence analysis was performed by omitting each study to find potential outliers [20]. The two authors inputted the data in the statistic software programs STATA version 11.0 to perform the statistical analysis independently and got the same results.

## Results

### Extraction process and study characteristics

**Figure 1:**
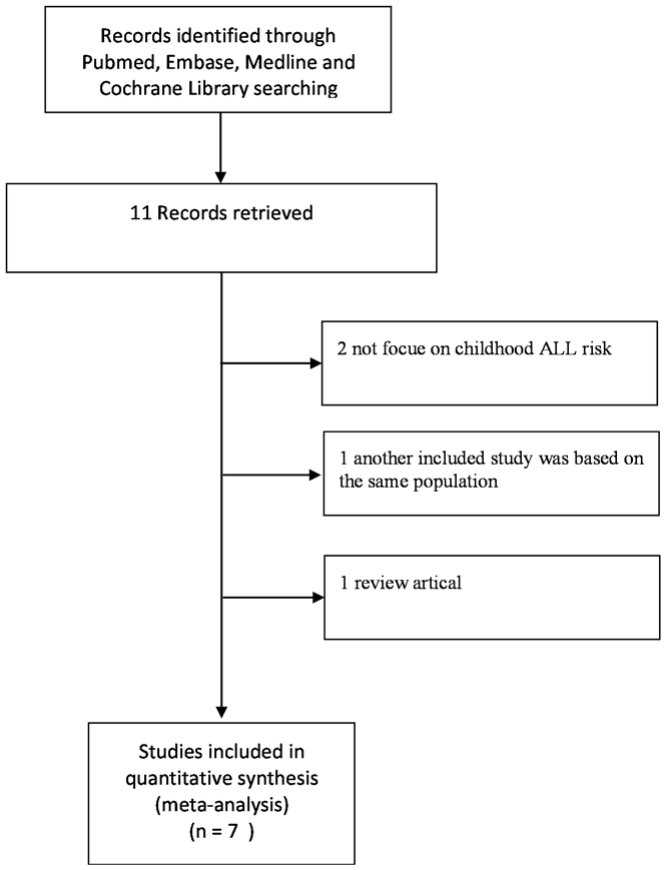
Flowchart of selection of studies for inclusion in meta-analysis.

With our search criterion, a total of eleven full-text articles [4], [9]–[15], [21]–[23] were preliminarily identified for further detailed evaluation (Fig. 1). Two studies that not focused on childhood ALL risk were excluded after title review. One study [23] was excluded as another included study [13] was based on the same population, and one was a systematic review. At last, seven case-control studies [9]–[15] were selected, including a total of 880 cases and 1311 controls originally. A list of characteristics of these included studies was provided in Table 1. There were 7 studies with a total of 880 cases and 1311 controls for Arg399Gln polymorphism, 3 studies with a total of 345 cases and 554 controls for Arg280His polymorphism, and 6 studies with a total of 783 cases and 1180 controls for Arg194Trp polymorphism. Genotype distributions in the controls of all studies were in agreement with HWE except one [13].

**Table 1:** General characteristics of studies included in the meta-analysis.

Author	Year	Country	Ethnicity	Source of controls	Matching criteria	Case	Control	SNP studied	HWE^a^
Joseph	2005	India	Asian	Hospital-based	sex, age, ethnicity	117	117	194, 280, 399	Yes
Pakakasama	2007	Thailand	Asian	Hospital-based	ethnicity	108	317	194, 280, 399	Yes
Batar	2009	Turkey	Caucasian	Hospital-based	sex, age, ethnicity	70	75	194, 399	Yes
Meza-Espinoza	2009	Mexico	Mestizo	Hospital-based	ethnicity	120	120	194, 280, 399	Yes
Tumer	2010	Turkey	Caucasian	Hospital-based	ethnicity	167	190	194, 399	Yes^b^
Canalle	2011	Brizal	Mixed	Hospital-based	-	201	361	194,399	Yes
Stanczyk	2011	Poland	Caucasian	Hospital-based	ethnicity	97	131	399	Yes

a HWE Hardy–Weinberg equilibrium.

b Genotype distributions of Arg194Trp in the controls were significantly deviated from HWE 399 Arg399Gln, 194 Arg194Trp, 280 Arg280His.

### Meta-analysis results

#### Table 2 listed the main results of the meta-analysis

**Table 2:** Results of the meta-analysis on XRCC1 polymorphisms and childhood ALL risk.

Polymorphism	Analysis	Case/Control	Additive model(aa vs AA)		Dominant model(Aa+aa vs AA)		Recessive model (aa vs AA+Aa)	
			OR[95% CI]	*P*/*P*_het_	OR[95% CI]	*P*/*P*_het_	OR[95% CI]	*P*/*P*_het_
Arg399Gln	Overall	880/1311	1.501[1.112, 2.026]	**0.008/0.384**	1.316[1.104, 1.569]	**0.002/0.016**	1.324[0.998, 1.757]	0.052/0.628
	Asian	225/434	2.338[1.254, 4.359]	**0.008/0.903**	2.108[1.498, 2.967]	**0.000/0.821**	1.698[0.937, 3.077]	0.081/0.706
	Caucasian	334/396	1.289[0.830, 2.000]	0.258/0.168	1.275[0.945, 1.722]	0.112/0.381	1.138[0.760, 1.704]	0.530/0.228
	Mestizo	120/120	1.711[0.653, 4.479]	0.274/−	1.306[0.787, 2.169]	0.302/−	1.556[0.612, 3.954]	0.353/−
	Mixed	201/361	1.227[0.629, 2.397]	0.548/−	0.845[0.597, 1.194]	0.339/−	1.358[0.707, 2.606]	0.358/−
Arg280His	Overall	345/554	1.251[0.371, 4.220]	0.709/0.675	1.125[0.805, 1.574]	0.490/0.534	1.203[0.357, 4.055]	0.765/0.693
	Asian	225/434	1.053[0.251, 4.426]	0.944/0.425	1.172[0.774, 1.775]	0.453/0.284	0.989[0.234, 4.175]	0.988/0.461
	Mestizo	120/120	2.023[0.180, 22.720]	0.568/−	1.043[0.590, 1.843]	0.885/−	2.017[0.180, 22.545]	0.569/−
Arg194Trp	Overall	783/1180	0.806[0.451, 1.438]	0.465/0.041	1.056[0.850, 1.312]	0.625/0.059	0.797[0.445, 1.426]	0.444/0.070
	Asian	225/434	0.710[0.319, 1.578]	0.400/0.014	0.966[0.684, 1.363]	0.843/0.007	0.714[0.319, 1.600]	0.414/0.028
	Caucasian	237/265	0.585[0.145, 2.351]	0.450/0.056	1.240[0.781, 1.969]	0.361/0.166	0.551[0.136, 2.233]	0.404/0.061
	Mestizo	120/120	2.150[0.520, 8.885]	0.290/−	1.265[0.730, 2.190]	0.402/−	2.053[0.501, 8.405]	0.317/−
	Mixed	201/361	0.443[0.049, 4.000]	0.469/−	0.929[0.586, 1.474]	0.755/−	0.446[0.050, 4.020]	0.472/−

OR Odds ratio; 95% CI 95% confidence interval.

a:minor allele; A:major allele.

**Figure 2:**
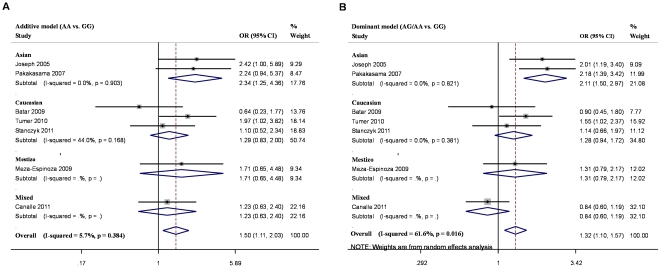
Meta-analysis of XRCC1 Arg399Gln polymorphism in childhood ALL. a Additive model, b Dominant model.

The results of the associations between *XRCC1* Arg399Gln polymorphism and childhood ALL risk, and the heterogeneity test were shown in Table 2. When all the eligible studies were pooled into the meta-analysis, elevated childhood ALL risk was revealed in additive model (OR = 1.501, 95% CI 1.112–2.026, P_OR_ = 0.008, P = 0.384 for heterogeneity)(Fig. 2a). We also found a significant association with childhood ALL risk in the dominant model (OR = 1.316, 95% CI = 1.104–1.569, P_OR_ = 0.002, P = 0.016 for heterogeneity) (Fig. 2b). No significant association was found in the recessive model (OR = 1.324, 95% CI = 0.998–1.757, P = 0.628 for heterogeneity). In subgroup analysis by ethnicity, the results revealed significant associations between the *XRCC1* Arg399Gln polymorphism and childhood ALL in Asian population (Additive model: OR = 2.338, 95%CI = 1.254–4.359, P_OR_ = 0.008, P = 0.903 for heterogeneity; Dominant model: OR = 2.108, 95%CI = 1.498–2.967, P_OR_ = 0.000 P = 0.821 for heterogeneity). We did not observe any significant association in any genetic model among other subgroups. Moreover, meta-regression analysis revealed that ethnicity was a significant source of between-study heterogeneity (P = 0.007).

There was no statistical difference in all contrasts of genotypes for Arg280His polymorphism (Additive model: OR = 1.251, 95%CI 0.371–4.220, P_OR_ = 0.709; Dominant model: OR = 1.125, 95% CI 0.805–1.574, P_OR_ = 0.490; Recessive model: OR = 1.203, 95%CI 0.357–4.055, P_OR_ = 0.765) and Arg194Trp polymorphism(Additive model: OR = 0.806, 95% CI 0.451–1.438, P_OR_ = 0.465; Dominant model OR = 1.056, 95% CI 0.850–1.312, P_OR_ = 0.625; Recessive model = 0.797, 95% CI 0.445–1.426, P _OR_ = 0.444). Subgroup analysis based on ethnicity also showed no significant association between the two SNPs and childhood ALL risk.

### Tests of heterogeneity

We have found heterogeneities in four studies: Arg399Gln polymorphism dominant model (p = 0.016, *I* ^2^ = 61.6%); Arg194Trp polymorphism additive model (p = 0.041, *I* ^2^ = 56.9%), dominant model (p = 0.059, *I* ^2^ = 53%), recessive model (p = 0.070, *I* ^2^ = 50.9%) (Table 2). A random-effects model was employed in these studies.

### Sensitivity analysis

**Figure 3:**
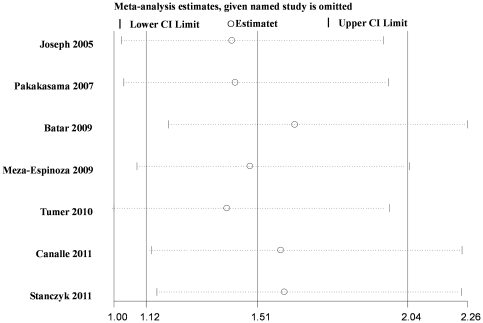
Influence analysis for AA versus GG in the overall meta-analysis. This figure shows the influence of individual studies on the summary OR. The middle vertical axis indicates the overall OR and the two vertical axes indicate its 95% CI. Open circles indicate the pooled OR when the left study is omitted in this meta-analysis. The two ends of the dotted lines represent the 95% CI.

Influence analysis was performed to assess the influence of each individual study on the pooled OR by sequential omission of individual studies. The results suggested that no individual study significantly affected the pooled ORs (Fig. 3).

### Publication bias

**Figure 4:**
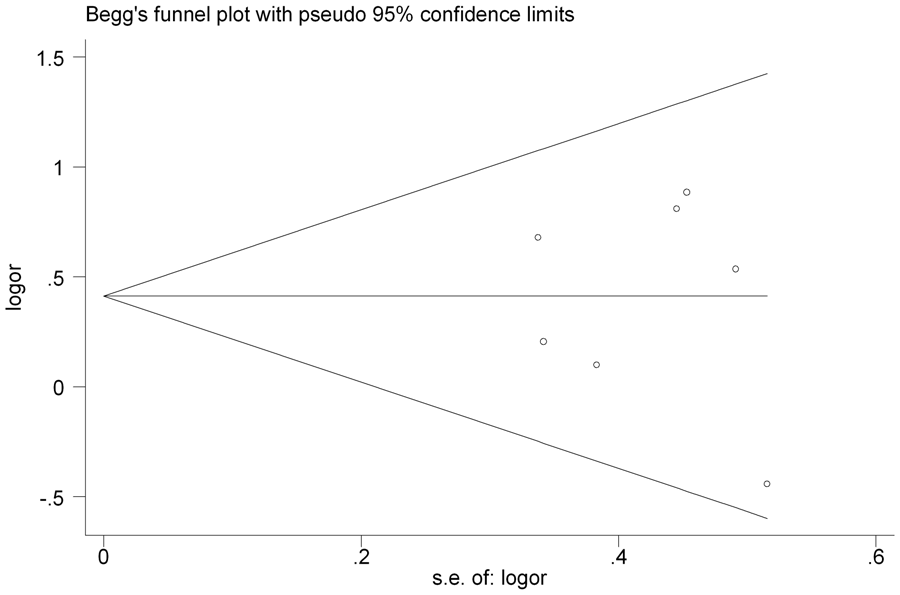
Funnel plot of XRCC1 Arg399Gln polymorphism and childhood ALL risk for publication bias.

Publication bias was examined by Funnel plot and Egger's regression test. The shapes of the funnel plot did not indicate any evidence of obvious asymmetry in additive model (Fig. 4) and the Egger's test suggested the absence of publication bias (P = 0.810).

## Discussion

*XRCC1* plays an important role in the DNA damage repair pathway for the processing of small base lesions, which has been thought of as the predominant DNA-damage repair pathway for the processing of small base lesions derived from oxidation and alkylation damage [7]. It is widely accepted that alterations in DNA repair genes play roles in the process associated with the etiology of cancers. In some of the previous studies, it has been reported that carriers of the variant allele were at higher risk of lung cancer [24], breast cancer [25], and prostate cancer among Asians [26], whereas the result was controversial in gastric cancer [27], as well as in bladder caner [28]. In this meta-analysis, we focused on *XRCC1* genetic polymorphisms and provide the most comprehensive assessment of its association with childhood ALL risk. By critically reviewing 7 studies on *XRCC1* Arg399Gln polymorphism (a total of 880 cases and 1311 controls), 3 studies on *XRCC1* Arg280His polymorphism (a total of 345 cases and 554 controls), and 6 studies on Arg194Trp polymorphism (a total of 783 cases and 1180 controls), we performed a meta-analysis to indicate that the polymorphisms in *XRCC1* Arg399Gln was significantly associated with risk of childhood ALL. However, we did not observe associations of *XRCC1* Arg280His polymorphism and Arg194Trp polymorphism with childhood ALL risk.

This study showed that the mean frequency of the *XRCC1* variant 399Gln allele was 28.99% (Table S1). Previous investigations found that the frequency distribution of 399Gln allele significantly varied in different ethnicities. Among Asian population, 22%∼28% had at least one copy of the variant allele *XRCC1* 399Gln [9]–[10], [29]–[30], while among Caucasian, Turkish, and other ethnic populations, the frequencies were 28%∼43% [11], [13], [31]–[33]. This may lead to *XRCC1* Arg399Gln polymorphism genotype distribution disequilibrium when all ethnic populations were pooled together. Ethnicity was significantly associated with childhood ALL risk and it was considered as a significant source of heterogeneity in the meta-regression model. It was essential to conduct a subgroup analysis based on ethnicities. In this meta-analysis, all subjects were classified into four ethnic groups (Caucasian, Asian, Metizo and mixed populations). No association was detected in Caucasians, Metizo and mixed populations, while increased risk was found in Asian population carrying variant 399Gln allele homozygote, and it should be further investigated in large scale Asian populations.

Despite lots of investigations in many aspects of childhood ALL, little attention has been paid to its pathogenesis, particularly with respect to genetic susceptibility. Several reports have demonstrated the association between some DNA repair gene variants and childhood ALL, so the possible relationship between polymorphisms of DNA repair genes and childhood ALL may be helpful in understanding the pathogenesis of childhood ALL and the prevention of this disease. Our results suggest that the risk of childhood ALL may be associated with DNA repair mechanisms. XRCC1 polymorphisms may be used as an important predictive factor, and ethnic background might have an impact on the results in the studies of its polymorphisms in childhood ALL. Analysis of these polymorphisms, particularly XRCC1 codon 399 Arg/Gln may help in identifying individuals at risk of developing ALL and providing an essential information source for future improvement of ALL treatment.

Although our result is suggestive, there are still some limitations in this meta-analysis. First, heterogeneity among the studies, resulting from different sources of controls, matching criteria of age and gender or some other factors, may influence the results of this analysis. The matching criteria of the control group, such as age, gender, and environment exposures, are different between studies. Furthermore, childhood ALL is heterogeneous considering its underlying cellular and molecular biology, so subtypes of B- or T-cell precursor ALL may not be suspected to share a common etiology. To date none of the studies have examined the relationship between *XRCC1* variants and risk by subtype. Second, specific environmental and lifestyle factors may alter those associations between gene polymorphisms and cancer risk. Evidence supports that there is no association between Gln/Gln genotype of Arg399Gln and bladder cancer risk in total population, but it is associated with a decreased risk of bladder cancer among ever smokers [34], [35]. The relationship between *XRCC1* gene polymorphism and childhood ALL risk was analyzed without consideration of gene–gene and gene-environment interactions because of the lack of sufficient data, which should be further investigated. Third, the Funnel plot did not reveal any evidence of obvious publication bias, while there is still a possibility that our meta-analysis was biased toward a positive result since negative findings were likely to be unreported.

In summary, it is a worthy and meaningful enterprise to search for polymorphic variants influencing the risk of childhood ALL. This meta-analysis suggests that *XRCC1* 399Gln might be a susceptibility allele for childhood ALL. However, we could not observe any association of *XRCC1* Arg194Trp and Arg280His with childhood ALL. We have searched as many publications as we could by means of various searching approaches, comprehensively assessed publication biases and pinpointed the potential sources of heterogeneity via subgroup and sensitivity analysis. However, these results may be biased by the relatively small number of subjects, and therefore need to be validated by larger studies and subsequent update of the current meta-analysis. In order to understand the mechanisms underlying childhood ALL better, future research should be considered and investigated.

## Supporting Information

Table S1 **Frequencies of XRCC1 Arg194Trp, Arg280His and Arg399Gln allele among control population in different studies.**
(DOC)
