# Supplementary material for: X-Ray Repair Cross-Complementing Group 1 (XRCC1) Genetic Polymorphisms and Risk of Childhood Acute Lymphoblastic Leukemia: A Meta-Analysis
Source: PLoS One. 2012 Apr 18;7(4):e34897. doi: 10.1371/journal.pone.0034897 (PMC3329555; doi:10.1371/journal.pone.0034897)
Supplement: Table S1 — Frequencies of XRCC1 Arg194Trp, Arg280His and Arg399Gln allele among control population in different studies. (DOC) [file pone.0034897.s001.doc]

Table S1. Frequencies of XRCC1 Arg194Trp, Arg280His and Arg399Gln allele among control population in different studies.

| Author | Year | Ethnicity | Arg194Trp | | Arg280His | | Arg399Gln | |
| --- | --- | --- | --- | --- | --- | --- | --- | --- |
|  |  |  | Arg194 | 194Trp | Arg280 | 280His | Arg399 | 399Gln |
| Joseph | 2005 | Asian | 0.87 | 0.13 | 0.85 | 0.15 | 0.78 | 0.22 |
| Pakakasama | 2007 | Asian | 0.70 | 0.30 | 0.92 | 0.08 | 0.75 | 0.25 |
| Batar | 2009 | Caucasian | 0.93 | 0.07 | - | - | 0.57 | 0.43 |
| Meza-Espinoza | 2009 | Mestizo | 0.85 | 0.15 | 0.86 | 0.14 | 0.74 | 0.26 |
| Tumer | 2010 | Caucasian | - | - | - | - | 0.69 | 0.31 |
| Canalle | 2011 | Mixed | 0.91 | 0.09 | - | - | 0.73 | 0.27 |
| Stanczyk | 2011 | Caucasian | 0.91 | 0.09 | - | - | 0.60 | 0.40 |
